# Primary meningeal central nervous system lymphoma: A case report and literature review

**DOI:** 10.1097/MD.0000000000032567

**Published:** 2022-12-30

**Authors:** Xue Chen, Min Huang, Zhenyuan Zhang, Huilan Jing, Yueli Zou, Hui Bu

**Affiliations:** a Department of Neurology, The Second Hospital of Hebei Medical University, Shijiazhuang, China; b Department of Neurology, Yuncheng Central Hospital of Shanxi Province, Shanxi, China.

**Keywords:** cerebrospinal fluid, cytopathology, flow cytometry, meninges, primary central nervous system lymphoma

## Abstract

Primary central nervous system lymphoma (PCNSL) is a rare extranodal non-Hodgkin lymphoma, and isolated meningeal PCNSL, without evidence of parenchymal involvement, is even less common, occurring in only 10% to 15% of cases.

**Patient concerns:**

A 65-years-old female presented to our hospital with progressive lower extremity motor dysfunction and blurred vision. The initial neurological examination revealed decreased muscle strength in both lower extremities and sensory dysfunction of lower extremities, saddle area, and buttocks. Brain magnetic resonance imaging showed no abnormalities. Lumbar enhanced magnetic resonance imaging showed T11 to L3 horizontal meningeal enhancement. Cerebrospinal fluid (CSF) cytology revealed lymphoma cells. Immunohistochemistry and flow cytometry of the CSF were performed as auxiliary methods to establish the diagnosis of lymphoma.

**Diagnoses:**

The patient was diagnosed primary meningeal central nervous system lymphoma.

**Interventions:**

During hospitalization, the patient was treated with 2 courses of high-dose intrathecal methotrexate and rituximab combined with intrathecal chemotherapy and supportive treatment.

**Outcomes:**

After 2 years of follow-up, the patient was able to walk and take care of herself.

**Lessons:**

Cases of PCNSL involving only the meninges are rare. Multimodal analysis of the CSF comprises an important component of the diagnostic work-up for patients with primary meningeal central nervous system lymphoma.

## 1. Introduction

Primary central nervous system lymphoma (PCNSL) is a rare aggressive extranodal non-Hodgkin lymphoma, accounting for 3% of all non-Hodgkin lymphomas and 3% of all primary central nervous system tumors.^[1]^ Immunocompromised individuals are at great risk of developing the disease; however, the incidence of PCNSL is also increasing in immunocompetent populations.^[2]^ The annual incidence of the disease ranges from 0.4 to 0.5 per 1,00,000.^[3,4]^ Although PCNSL can develop at any age, the median age of patients at diagnosis is 65 years, and the incidence in the elderly population has been rising.^[5,6]^ PCNSL with meningeal involvement only is rare. In this paper, we report a case of primary meningeal lymphoma, in which the patient was finally diagnosed by cerebrospinal fluid cytology, cerebrospinal fluid immunocytochemical staining and flow cytometric analysis, and this case study aims to further investigate the basis and significance of cerebrospinal fluid analysis as a modern “fluid biopsy” in the diagnosis of primary meningeal central nervous system lymphoma (PMCNSL).

## 2. Case report

A 65-years-old female suffered from progressive motor dysfunction in her lower extremities, which arose 4 months prior to the assessment. Initially, she could walk but had paroxysmal dizziness and blurred vision. Her past medical history was unremarkable, and she had no evidence of predisposing immunodeficiency. The ultrasound examination results and the peripheral blood tests, including tests for the human immunodeficiency virus, Epstein–Barr virus and lymphopenia, were either normal or negative.

Neurological examination revealed mildly decreased muscle strength, the disappearance of tendon reflexes in both lower extremities, and the loss of sensory function in the saddle area, buttocks, and lower extremities. The rest of the physical examination was unremarkable. No swelling of superficial lymph nodes was observed at any location. Her lumbar spinal computed tomography (CT) performed at the local hospital showed only lumbar disc herniation and bone hyperplasia. She was clinically diagnosed with “lumbar disc herniation.” After treatment, no relief was obtained. Meanwhile, the patient had difficulty standing and walking, accompanied by weakness in the left upper extremity. A subsequent lumbar puncture showed an increase in aberrant lymphocytes in the cerebrospinal fluid (CSF).

**Figure 1.**
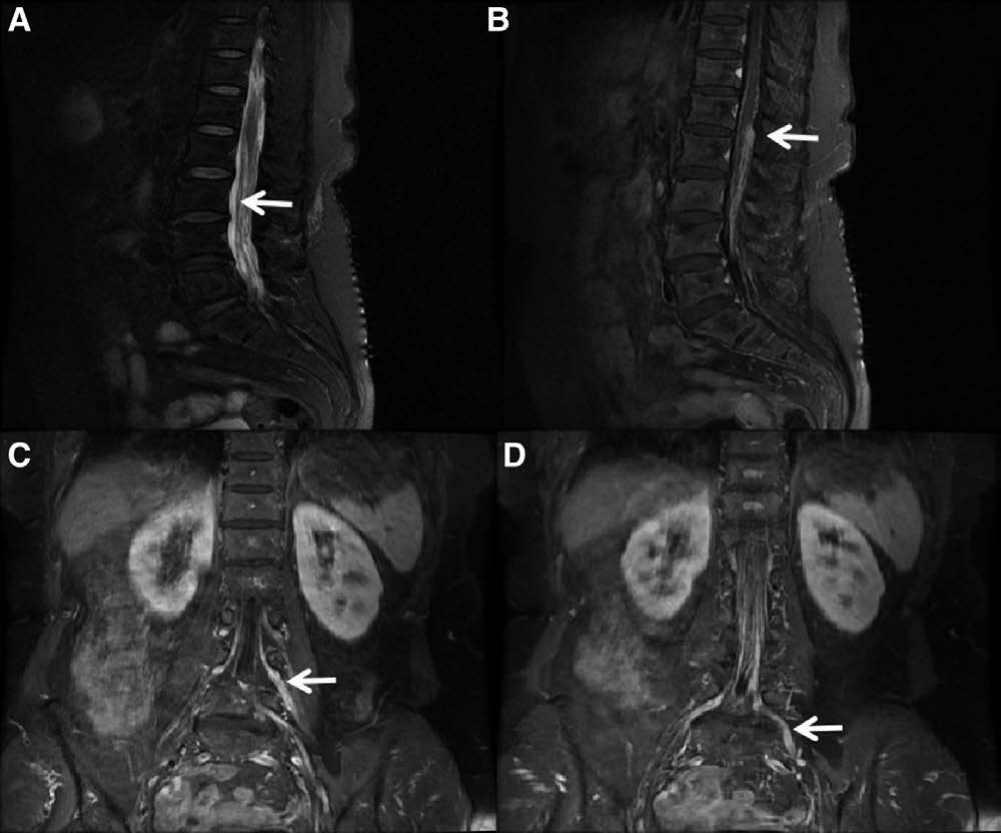
Imaging. (A) Contrast lumbar spine MRI demonstrates multiple abnormal enhancements in the spinal canal of the lower part of the thoracic vertebrae in the 10-lumbosacral region, mainly within the dura mater and terminal filaments. (B) The lumbar 1–2 intervertebral space level of the right spinal has nodule-like enhancement, the border of which is not vague, and the range is approximately 0.4 cm × 0.6 cm × 1.0 cm. (C&D) Enhancement can be seen along the nerve root in the lumbar 4- sacral 1 intervertebral foramen. MRI = magnetic resonance imaging.

The patient was then transferred to our hospital. After admission, the Gadolinium-enhanced magnetic resonance imaging (MRI) of the thoracic and lumbar spine further demonstrated the enhancement of the dura mater, the terminal filaments and some nerve roots of the lumbosacral region, and there was enhancement of the thoracic cord leptomeninges (Fig. 1). The MRI findings showed lesions in the dura mater and leptomeninges, and these findings indicated a malignant tumor.

**Figure 2.**
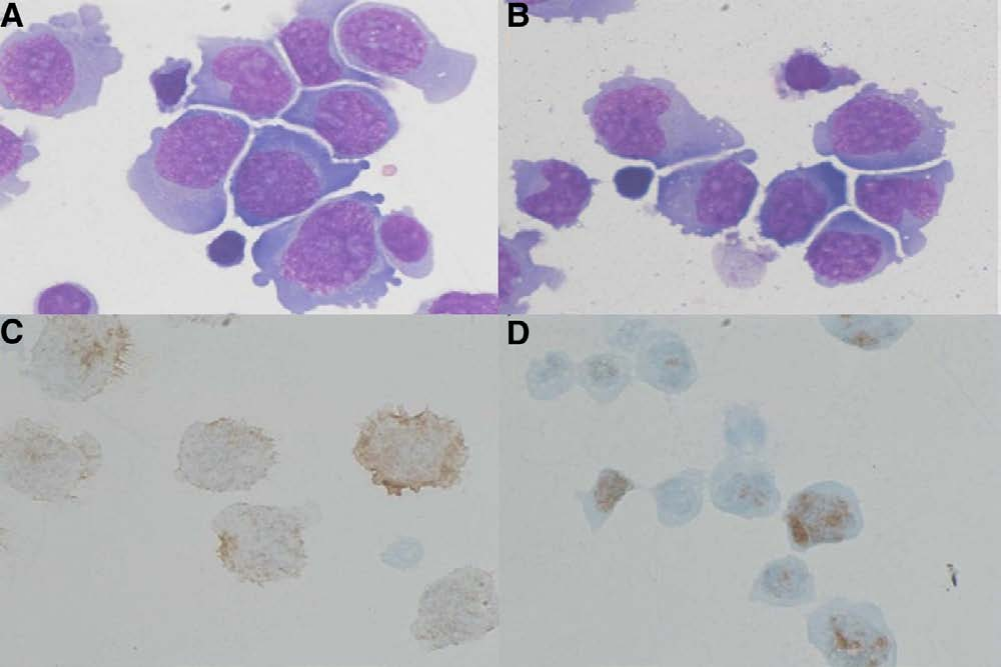
Cerebrospinal fluid cytology and CSF immunohistochemistry. (A, B) It can be seen that cells with large size, large nuclei, coarse nuclear chromatin, imbalanced cytoplasmic ratio, basophilic cytoplasm, and morphology and characteristics are similar to malignant cells (MGG × 1000). (C) CSF immunohistochemistry showedCD20 (+), (D)Ki67 (+). CSF = cerebrospinal fluid.

**Figure 3.**
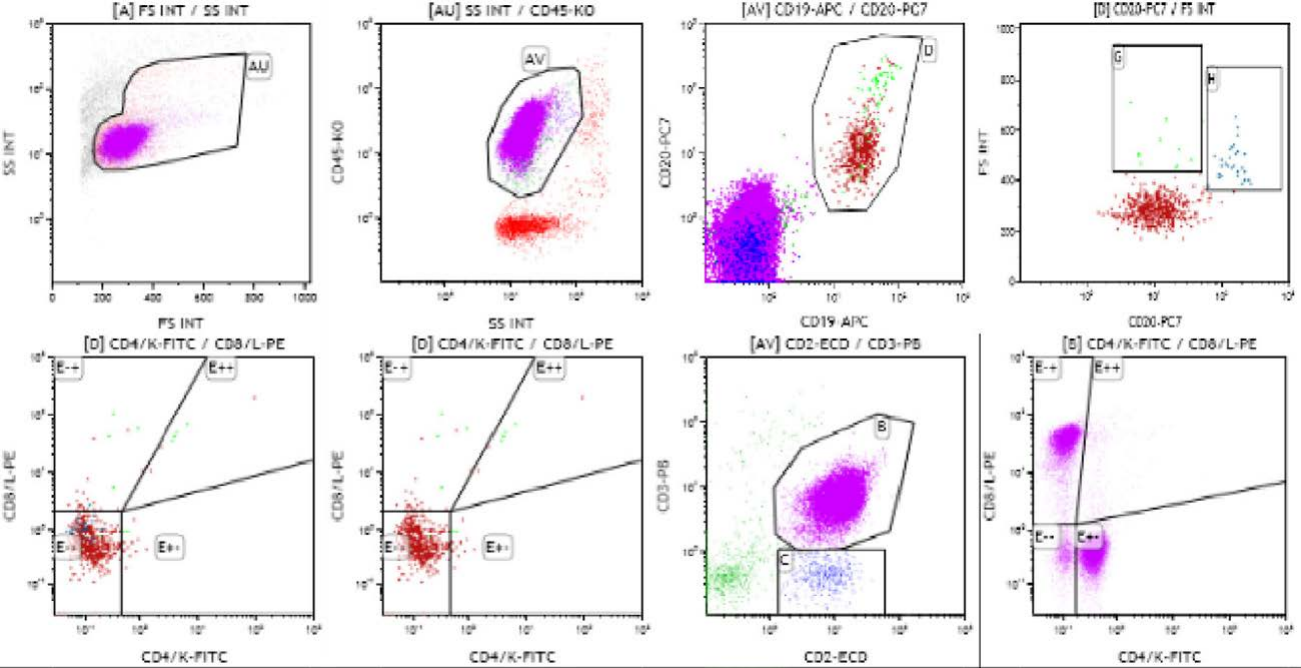
CSF flow cytometry. Using CD45/SSC as the gate; a total of 27800 nucleated cells were analyzed. The percent of CD20^ + ^CD19^ + ^lymphocytes was approximately 2.19%, without expression of CD10, and the surface *κ*/*λ* ratio was normal (except for a few large cells in the G gate, which expressed a single *λ* light chain). No significant abnormality was detected in the T phenotype. CSF = cerebrospinal fluid.

**Figure 4.**
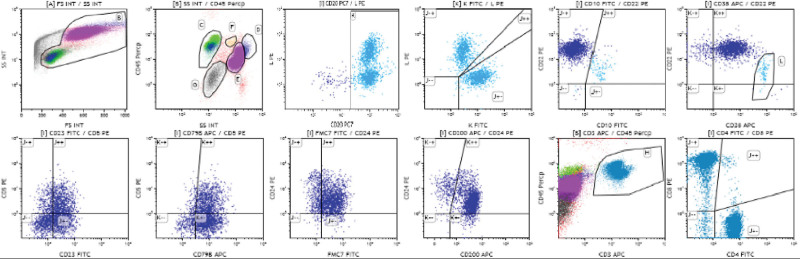
Bone marrow fluid flow cytometry. Within the nucleated cells, the percent of CD19 + lymphocytes accounted for approximately 3.61%, and their phenotypes included strongly expressed CD22, CD10-CD22 + (94.9%), CD10 + CD38 + (4.26%), CD20 (95.3%), which expressed *κ* (53.9%) and *λ* (44.5%) on the surface), CD23 (63.9%), CD5 (41.5%), CD79b (85.8%), FMC7 (79.9%), CD24 (92.2%) and CD200 (87.1%). A small amount of weak fluorescent expression of CD11c and CD25 was observed in all CD19^ + ^cells, and no cells showed significant expression of CD103.

Because atypically proliferating lymphocytes were found in the CSF by a prior cytopathological exam, a subsequent lumbar puncture was immediately performed, and we found that the white blood cells in the CSF were increased, in which the monocyte percent was 90.00%. CSF cytology revealed malignant lymphoma cells (Fig. 2). On CSF immunohistochemistry, a population of Ki67- and CD20-positive cells with monotypic expression was found (Fig. 2). According to the morphology and immunohistochemical results of the malignant cells found in CSF, considering the possibility of lymphoma, CSF flow cytometry (FCM) studies reported a small number of large B cells expressing single lambda immunoglobulin light chains, and no significant abnormality was detected in the T phenotype (Fig. 3). This unusual presentation prompted a work-up for systemic lymphoma. The sample submitted for bone marrow aspiration biopsy results revealed that hematopoietic cells, including the 2 lines of granulocytes and erythrocytes, were visible, and no megakaryocytes were seen. Bone marrow FCM demonstrated a small number of primary and early B lymphocytes within the nucleated cells, and the measured T and mature B lymphocytes showed no abnormalities (Fig. 4). Both bone marrow results implied malignancy negativity. Whole-body positron emission tomography (PET-CT) showed increased metabolism in the cerebellar meningeal region and inferior nerve root, providing no evidence for extracranial or primary nodal disease. Ultimately, a diagnosis of primary meningeal central nervous system lymphoma was made based on the above findings.

Due to the special location of the lesion and the neurosurgeon’s consultation, it is difficult to obtain a biopsy of the meninges, so we regret that we still have not performed a biopsy of the lesion.

After the diagnosis was confirmed, a total of 6 rounds of chemotherapy were performed, and high-dose methotrexate (3 mg/m2 4.89 g d2) and rituximab (375 mg/m2 600 mg d1) were intravenously injected, combined with an intrathecal injection of “methotrexate 10 mg + dexamethasone 10 mg” and supportive treatment. At a follow-up in January 2022, the patient showed significant improvement in her symptoms of blurred vision and limb weakness, could walk, could take care of herself, and had no assistance from others. Cerebrospinal fluid cytology was negative.

## 3. Discussion

PCNSL is a rare and aggressive extranodal non-Hodgkin lymphoma that refers to a disease that originates from and localizes to the brain, eyes, spinal cord, or meninges without systemic involvement.^[7]^ The majority of PCNSL cases are diffuse large B-cell lymphoma, accounting for approximately 95%, and the rest are composed of T cells (2%), Burkitt, lymphoblasts, and low-grade lymphomas.^[1]^ PMCNSL is rare, and few related cases have been described. Its clinical manifestations are nonspecific, and patients may present with increased intracranial pressure, headache, vomiting, seizures, meningeal irritation signs, that is, or with symptoms such as cranial nerve palsy and spinal nerve root irritation pain.

Currently, the diagnosis of PMCNSL remains challenging. Pathological tissue biopsy is the gold standard for diagnosis whenever possible. However, because the lesions mainly involve the meninges, spinal arachnoid membrane and peripheral nerve roots and are mostly diffuse, tissue biopsy for PMCNSL is often not easy to perform. Finally, we failed to obtain a pathological biopsy of the diseased tissue in this patient. Gadolinium-enhanced MRI is currently a more sensitive imaging modality for the diagnosis of PCNSL.^[8]^ Contrast-enhanced MRI may show marked thickening and enhancement of the meninges in the area where the lesion is located, or it may show minor focal abnormalities of the cranial nerves or nerve roots. Techniques such as 18F-fluorodeoxyglucose PET-CT can aid in the diagnosis of PMCNSL and can exclude systemic disease secondary to central nervous system involvement.^[9,10]^ In this patient, enhanced MRI of the thoracolumbar spine revealed multiple meningeal and partial nerve root enhancements in the thoracolumbar spine, and PET-CT showed increased metabolism of the meninges and some nerve roots at the cerebellar margin, both of which suggested possible malignant meningitis.

Multimodal analysis of the CSF comprises an important component of the diagnostic work-up for patients with suspected meningeal lymphoma, and these CSF analyses are more often selected for patients not suited for biopsy. Cytopathological examination of the CSF is generally considered the gold standard for the diagnosis of meningeal disease in PMNCSL.^[11]^ The cytological characteristics of lymphoma cells are enlarged cell size, basophilic cytoplasm, reticular chromatin, nucleoli, and mitosis with MGG staining. In practice, CSF cytology is relatively insensitive in the detection of primary CNS lymphoma, with only approximately 20% of diagnoses made in this manner. The low sensitivity is due to the morphological similarity between lymphoma cells in the CSF and benign reactive cells.^[12,13]^ Distinguishing normal or reactive lymphocytes from malignant lymphoma cells in cytomorphology is difficult.^[14]^ Experienced neuropathologists and repeated lumbar punctures are needed to improve sensitivity. In our case, repeated CSF cytologies demonstrated that cells with a large size, large nuclei, coarse nuclear chromatin, imbalanced cytoplasmic ratio, and basophilic cytoplasm were found, which was consistent with malignant lymphoma cells regarding morphology and characteristics.

The CSF immunohistochemical profile of primary meningeal central nervous system lymphoma is rarely reported. For lymphoma tissues, the majority of PCNSLs manifest with low expression of the marker CD10, expression of the marker BCL-6 and high expression of the activated B-cell-like marker MUM-1. Nuclear proliferation antigen (Ki67) is a nuclear antigen related to the specificity of cell proliferation, which can reflect the degree of cell proliferation, representing the active growth fraction of the tumor. Jing Liu et al^[15]^ reported that high Ki-67 expression was a valuable biological marker for poor prognosis by analyzing 89 patients’ biopsy tissues. In our patient, the CSF immunohistochemical profile demonstrated Ki67 positivity, implying the active growth of the lymphoma. The patient was positive for the marker CD20, indicating that the tested cells were B-cell lines responsive to rituximab therapy.

Quantitatively, when neoplastic cells constitute at least 5% of the nucleated cells, they can be detected by cytopathology. FCM enables the identification of B-cell clones that comprise as little as 0.2% of the total cellular number.^[12]^ Roland Schroers et al compared the cytopathology of conventionally stained slides with multiparameter FCM in CSF specimens collected from 30 PCNSL patients, revealing a higher sensitivity of FCM compared with cytopathology (23.3% vs 13.3%).^[16]^ Simone Canovi and his colleagues summarized that samples with positive FCM but had negative cytopathology were found in 89% (24/27) of the studies, with percentages ranging from 3.5% to 61.5%, while samples with positive cytopathology and negative FCM were found in 48% (13/27) of the studies with percentages ranging from 0.5% to 10%.^[17]^ These studies suggested that multiparameter FCM increases the sensitivity and specificity of leptomeningeal disease detection in CNS lymphomas.

The majority of PCNSL cases are diffuse large B-cell lymphomas, which are clonally restricted and express either kappa or lambda immunoglobulin light chains. Excessive *κ* and *λ* light chains are secreted to some extent as free immunoglobulin light chains (FLC), which are not bound to immunoglobulin heavy chains to form complete antibody molecules. Roland Schroers^[16]^ and their colleagues previously reported that the mean *κ*⁄*λ* FLC ratios in CSF samples were significantly (*P* = .04) higher in patients with lymphoma (33.9 ± 20.5) when compared to CSF samples of the control group (1.4 ± 0.2), while the difference in the *κ* or *λ* FLC concentrations in the CSF of CNS lymphoma patients and the CSF of control patients was not statistically significant, which just implies that the *κ* ⁄ *λ* FLC ratio in the CSF is a promising parameter in CNS lymphoma. Future studies will be needed to confirm it as a diagnostic tool in PNCSL. In our case, CSF FCM showed a small number of large B cells expressing single lambda immunoglobulin light chains, and no significant abnormality was detected in the T phenotype, implying that there were large B-cell clones.

In our case, malignant lymphoma cells were found in the cytology of the CSF, and spinal MRI demonstrated enhancement of the meninges. FCM and immunohistochemistry of CSF suggested the presence of clones of B lymphoma cells. Secondary central nervous system lymphoma was excluded by bone marrow aspiration biopsy and a whole-body PET-CT scan. The diagnosis of primary meningeal central nervous system lymphoma was finally established.

PCNSL has no recognized optimal therapy due to its slow onset and diffuse growth, high malignancy and easy recurrence. However, according to existing expert consensus in the field, high-dose methotrexate-based chemotherapy combined with multiple treatments is commonly used to curb the development of PCNSL.^[18]^ Combined with methotrexate, rituximab, which is a traditional chemotherapeutic agent and specifically targets CD20, has been widely used in systemic lymphoma. Rituximab combined with high-dose methotrexate showed a good complete response rate in the treatment of diffuse large B-cell lymphoma of the central nervous system.^[19]^

## 4. Conclusion

In conclusion, cases of primary central nervous system lymphoma involving only the meninges are rare. Early diagnosis is essential owing to the limited time frame to start potentially successful treatment. Sometimes it is not feasible to obtain a biopsy. Multimodal analysis of the CSF comprises an important component of the diagnostic work-up for patients with primary meningeal central nervous system lymphoma, and some auxiliary modalities, such as MRI or PET-CT, can improve the diagnostic sensibility and accuracy.

## Author contributions

**Conceptualization:** Xue Chen, Min Huang.

**Data curation:** Zhenyuan Zhang, Huilan Jing.

**Project administration:** Yueli Zou.

**Writing – original draft:** Xue Chen, Min Huang.

**Writing – review & editing:** Xue Chen, Min Huang, Hui Bu.
